# 480. Redefining the Epidemiologic Profile of Histoplasmosis from 2011 to 2021 in Central Ohio

**DOI:** 10.1093/ofid/ofac492.538

**Published:** 2022-12-15

**Authors:** JustIn Hofmann, Mohammad Mahdee Sobhanie, Medical Doctor, Sajed Sarwar, Courtney Hebert

**Affiliations:** Ohio State University Wexner Medical Center, Columbus, Ohio; The Ohio State University, Columbua, Ohio; The Ohio State University Wexner Medical Center, Columbus, Ohio; The Ohio State University, Columbua, Ohio

## Abstract

**Background:**

*Histoplasma* is a common endemic mycosis in multiple Midwestern states. Most of the data defining epidemiologic risk are from 20^th^ century outbreak investigations. A known exacerbating factor is immune suppression; but use of biologics, immunosuppressants (ISP), and chemotherapy has advanced since these investigations were conducted. This study sought to use clinical data from a tertiary care facility to address these gaps to define the evolving epidemiology of histoplasmosis in Ohio.

**Methods:**

In a single-center retrospective cohort study, we identified adult patients with a histoplasmosis-specific diagnosis, a positive diagnostic test, and an itraconazole/amphotericin B prescription between 1/1/2012 and 10/1/2021. Prisoners were excluded. A random sample of 100 patients underwent in-depth chart review. Comorbidities, environmental risk factors, and ISP use data were collected. Cases were classified by the CDC 2017 Histoplasmosis case definition. “Confirmed” cases had culture growth, a confirmatory lab result, or histopathology plus signs and symptoms of disease, and “probable” cases had a non-confirmatory lab result plus signs and symptoms. Associations between variables were assessed using Fisher’s exact test, p < 0.05.

**Results:**

Of 100 patients, 56 had confirmed and 22 had probable histoplasmosis (Table 1). Three had confirmed blastomycosis, 15 met definitions for both histoplasmosis and blastomycosis, and 4 met neither definition. These were excluded from analysis. No environmental exposure history was documented for 23 of 78 (29.4%) patients. Of those with documented history, 15 (27.1%) had recent renovation or construction exposure; 14 (25.4%) to soil, gardening, or outdoors; 12 (21.8%) to birds, bats, and farm animals; and 25 (45.4%) had no known exposure. There were 51 (65.4%) exposed to at least one dose of ISP in the year prior to diagnosis, including 41 (52.5%) to corticosteroids.

**Table 1:**
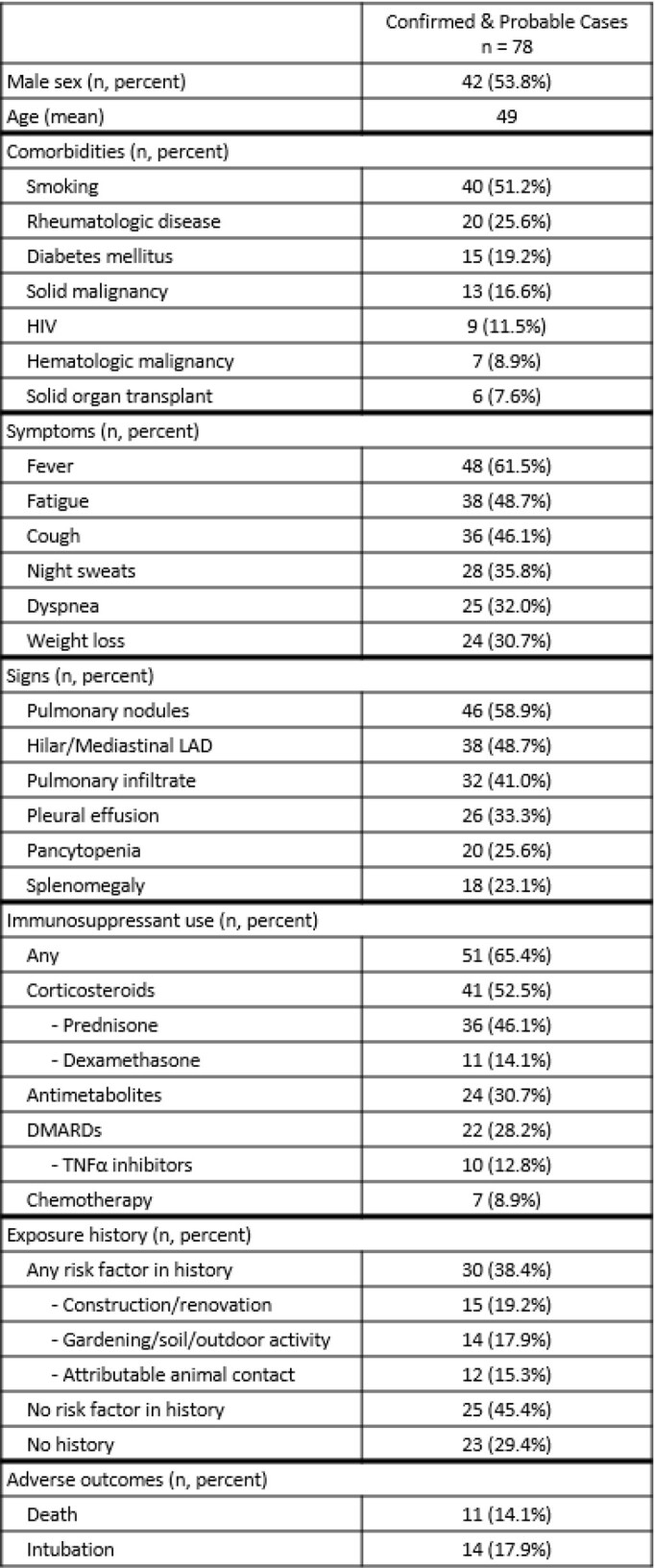
Characteristics of patient cohort

**Conclusion:**

In this cohort, more patients with confirmed or probable histoplasmosis were exposed to ISP than had a documented traditional environmental risk factor. This study shows reliance on traditional environmental exposure history might not accurately gauge risk. Further work is needed using ISP exposure to drive appropriate diagnostic testing.

**Disclosures:**

**All Authors**: No reported disclosures.

